# Identification of Non-Volatile Compounds Generated during Storage That Impact Flavor Stability of Ready-to-Drink Coffee

**DOI:** 10.3390/molecules27072120

**Published:** 2022-03-25

**Authors:** Hao Lin, Edisson Tello, Christopher T. Simons, Devin G. Peterson

**Affiliations:** Department of Food Science and Technology, Parker Food Science & Technology Building, The Ohio State University, 2015 Fyffe Rd., Columbus, OH 43210, USA; lin.1788@osu.edu (H.L.); tellocamacho.1@osu.edu (E.T.); simons.103@osu.edu (C.T.S.)

**Keywords:** ready-to-drink coffee, coffee flavor stability during storage, untargeted chemical profiling, degree of difference sensory evaluation, chlorogenic acids

## Abstract

Coffee brew flavor is known to degrade during storage. Untargeted and targeted LC/MS flavoromics analysis was applied to identify chemical compounds generated during storage that impacted the flavor stability of ready-to-drink (RTD) coffee. MS chemical profiles for sixteen RTD coffee samples stored for 0, 1, 2, and 4 months at 30 °C were modeled against the sensory degree of difference (DOD) scores by orthogonal partial least squares (OPLS) with good fit and predictive ability. Five highly predictive untargeted chemical features positively correlated to DOD were subsequently identified as 3-caffeoylquinic acid, 4-caffeoylquinic acid, 5-caffeoylquinic acid, 3-O-feruloylquinic acid, and 5-O-feruloylquinic acid. The increase in the six acidic compounds during storage was confirmed by sensory recombination tests to significantly impact the flavor stability of RTD coffee during storage. A decrease in pH, rather than an increase in total acidity, was supported to impact the coffee flavor profile.

## 1. Introduction

First introduced in the 15th century, coffee is a popular beverage that has become one of the most important agricultural commodities worldwide [1]. The value of the world coffee market is estimated to exceed USD 200 billion, and coffee consumption is steadily growing at an annual rate of 2.2% [2,3]. In 2019, the United States coffee market was valued at USD 15.6 billion and expected to grow 22.7% through 2024, driven in large part by the fast growth rate of ready-to-drink (RTD) products [4]. In general, the term RTD coffee encompasses shelf-stable or refrigerated bottled/canned coffee drinks. RTD coffee has become increasingly popular because of the growing demand for convenient beverage options [5]. It is forecasted that RTD coffee will surpass roasted coffee to become the largest segment in US coffee sales by 2024 [4].

Coffee and RTD coffee products are favored worldwide not only due to their stimulating effects but also their distinctive and pleasant flavor [6,7]. Consumer’s coffee purchase decisions are dependent on several factors, including functionality, packaging, branding, and sensory characteristics. Flavor has been identified as the most influential factor for purchase decisions [5,8]. As a result, the focus has been placed on the development of manufacturing processes and preservation strategies that deliver desirable, high-quality coffee flavors. For decades, the chemical basis and sensory properties of coffee flavor have been of research interest.

Coffee flavor is a complex combination of aroma, taste, and somatosensation [9]. Most research has focused on the discovery of key aroma and taste compounds that contribute to the flavor profile of coffee beans and brews [10,11,12,13,14]; however, literature specifically focused on the flavor of RTD coffee is limited. RTD coffee requires additional processing and storage and therefore faces challenges with flavor stability [15]. For example, the decrease in roasty odor notes of RTD coffee after heat processing has been attributed to the degradation of unstable odorants containing the thiol functional group [16,17]. Similarly, Murakami et al. [18] reported that the overall coffee flavor, coffee aroma, and bitterness of canned coffee drinks were weaker in intensity after the sterilization process. During storage, freshly prepared coffee brew packaged in aseptic glass bottles has been found to develop several unpleasant attributes such as rancid aroma, sourness, and an astringent aftertaste, as well as exhibit the loss of aroma intensity and freshness [19,20]; however, the connection between chemical changes and the corresponding impacts on sensory attributes of RTD coffee during storage is still limited. Moreover, from an analytical standpoint, less information has been reported regarding changes that occur in the non-volatile fraction of coffee brew during storage [19]. The lack of relevant knowledge opens the research opportunity to better understand RTD coffee flavor stability.

Traditionally, the discovery of flavor compounds in foods has relied on targeted approaches, which primarily focus on evaluating singular compounds in a unimodal sensory response [21,22,23]. However, these approaches can overlook the effects of contextual interactions and flavor modulators. In recent years, an untargeted flavor approach named flavoromics has been applied to understand the chemical drivers of flavor properties in complex food matrices [24,25,26]. Flavoromics combines comprehensive chemical profiling with statistical analysis to establish correlations between chemical components and sensory responses [27]. Using flavoromics, several tasteless compounds that modulated coffee flavor were discovered [28,29]. In addition, Ronningen et al. [30] identified the non-volatile flavor compounds that impacted the loss of orange freshness during aging.

This study aimed to apply untargeted flavoromics analysis to identify non-volatile chemical compounds that impact the flavor stability of RTD coffee during storage. In this phase of a two-part study, compounds that were generated during storage or positively correlated to flavor change were investigated. LC/MS chemical profiling with multivariate statistical analysis (MVA) was utilized to correlate RTD coffee compounds with overall flavor changes. Highly predictive markers were selected, purified, and identified, and their flavor relevance was confirmed by a sensory recombination experiment.

## 2. Results and Discussion

### 2.1. Sensory Evaluation of RTD Coffee Samples by Degree of Difference (DOD)

Two common coffee species (Arabic and Robusta) were prepared under two processing conditions, namely in air and under nitrogen, then aseptically processed, and stored over 4 months at 30 °C. The combination of sample conditions provided chemical and sensory data to support the discovery of general chemical drivers of RTD coffee flavor stability. In the current study, the RTD coffee samples were evaluated at hot temperatures between 60 to 65 °C during sensory evaluation for the following reasons. A hot serving temperature is preferred for coffee [31,32] with a long tradition of RTD coffee being sold as a hot beverage from vending machines or at convenience stores, especially in Japan where RTD coffee comprises the majority of coffee sales [33]. Additionally, the basic taste perceptions are known to be the most sensitive around room temperature (30 °C) and exhibit higher threshold values as temperatures increase or decrease [34,35]. Hence, any taste-based flavor changes that were noted in hot RTD coffee, would also be expected to be noticeable when RTD coffee is consumed at room temperature.

The flavor changes of RTD coffee during storage were measured by a degree of difference (DOD) test and shown in Table 1. It could be seen that all aged RTD coffee samples (1 month, 2 month, and 4 month) were found to be significantly different (*p* < 0.05) from the non-aged samples (control), while not all the aged samples were found to be significantly different from each other. For example, the DOD scores for the specific Arabica nitrogen-flushed RTD coffee for the blind control (non-aged), 1-month, 2-month, and 4-month aged samples were 1.2, 4.4, 5.6, and 6.6, respectively. No significant difference was observed between 1-month and 2-month samples, as well as between 2-month and 4-month samples. These results indicated that the largest flavor changes occurred between the non-aged and the 1-month aged sample, and the flavor changes increased over the 4 months of storage. A similar trend of changes was observed in the DOD scores over time for the RTD coffees varieties from different coffee species and processing conditions.

In addition to DOD scores, panelists (*n* = 14) provided qualitative comments on the flavor differences observed between RTD coffee samples. In general, panelists indicated that sourness was one of the major drivers for the DOD scores. For example, “more sour” was mentioned 12 times, followed by “more fruity” 6 times, “more astringent” 3 times, and “less coffee aroma” 3 times for the 4-month aged Arabica nitrogen-flushed sample. Given that sourness is known to be impacted by the pH and acidity in coffee [36], the pH values of RTD coffee samples were measured. The non-aged coffee decreased from pH 4.92 to 4.76 in the 4-month aged Arabica nitrogen-flushed RTD coffee sample; similar results were observed in other RTD coffee samples. This observation was in agreement with previous studies that reported a decrease in pH and the development of sour taste in coffee brew during 60 days of storage [19,37]. Anese and Nicoli [38] reported a zero-order kinetic change in [H^+^] concentration in RTD coffee during storage and suggested that the rate of pH decrease was not affected by the presence of oxygen. Thus, in the current study, it was expected that the noted increased sour attributes and reduction in pH values during storage would originate from the generation of acidic compounds.

To further investigate the compounds that contributed to the overall flavor changes of aged RTD coffee and noted development of sour taste, DOD scores were modeled with chemical data in the multivariate analysis.

### 2.2. Multivariate Statistical Modeling

**Figure 1:**
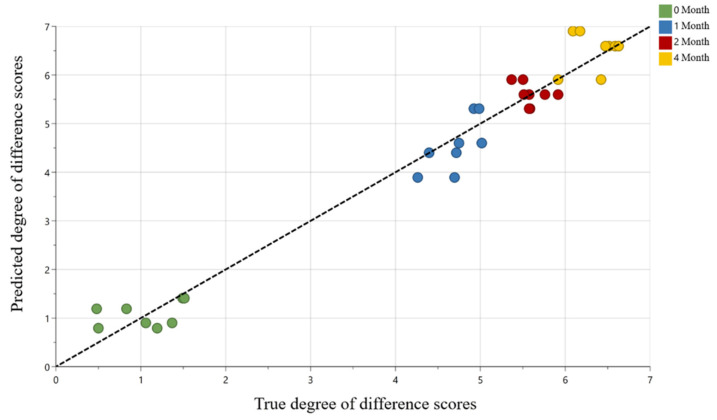
Regression plot of the predicted versus true degree of difference (DOD) scores from the OPLS model (Pareto scaling) correlating LC/MS chemical profiling from 16 RTD coffee samples in biological replicate. OPLS model quality values were R^2^Y = 0.966 and Q^2^ = 0.960 with a root mean squared error of prediction of 0.4 out of a total of 10 points. Coffee samples color-coded by storage time.

**Figure 2:**
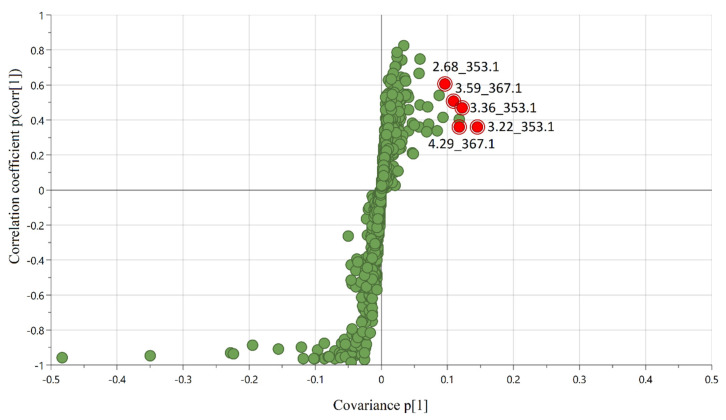
S–plot from OPLS model (Pareto scaling) correlating DOD scores and LC/MS chemical profiling from 16 RTD coffee samples in biological replicate; highlighted dots represent the selected positively correlated chemical markers (retention time_*m*/*z*) of interest with VIPpred score of 3.7–5.6.

Compounds that correlated with flavor change in RTD coffee during storage were modeled using a total of 1489 LC/MS chemical features against the DOD scores. An initial unsupervised principal component analysis (PCA, data not shown) confirmed that high-quality chemical data were obtained from UPLC-MS profiling (R^2^X = 0.926, Q^2^ = 0.887). Subsequently, a supervised orthogonal partial least squares (OPLS) model was built to investigate the contribution of chemical features to the DOD scores. The high model predictive power was further illustrated in Figure 1 (R^2^X = 0.912, R^2^Y = 0.966, Q^2^ = 0.960). Predicted DOD scores from the OPLS model were plotted against the true DOD scores, showing a root mean squared error of prediction (RMSEP) of 0.4 out of a total of 10 points. To investigate sensory-active compounds generated during storage, the five most predictive chemical features based on the variable of importance scores (VIPpred) that were positively correlated were selected. The VIP score value indicates the contribution of X variables (chemical features) in predicting the Y variable (DOD scores). Generally, X variables with VIP value > 1 are considered significant contributors [39]. The five positively correlated features were among the top ten predictive features for the OPLS model with a VIPpred score of 3.7–5.6 and are shown in Figure 2 and reported in Table 2 (compounds **1**–**5**).

### 2.3. Identification of Positively Correlated Predictive Compounds

Compounds **1**, **2**, and **3**, with VIPpred scores of 3.7, 5.6, and 4.7, respectively (Table 2), displayed the same accurate mass parent ion [M–H]^–^ at *m*/*z* 353.0882, which corresponds to a molecular elemental composition of C_16_H_18_O_9_ (∆mass = 0.67 ppm), indicating the three compounds were isomers. MS/MS fragmentation also revealed a common product ion of *m*/*z* 191.1 after losing 162.0 mass units. This fragmentation pattern matched the cleavage of an ester bond between quinic acid and caffeic acid moieties in a caffeoylquinic acid molecule [40]. Hence, features 2.68_353.1, 3.22_353.1, and 3.36_353.1 (Table 2) were compared to authentic commercial standards by matching retention time, accurate mass, and MS/MS fragmentation (see Supplemental Materials, Figure S1); and compounds **1**, **2**, and **3** were identified as 3-caffeoylquinic acid (3-CQA), 5-caffeoylquinic acid (5-CQA), and 4-caffeoylquinic acid (4-CQA), respectively (Table 2).

Compounds **4** and **5** with VIPpred scores of 4.1 and 4.5, respectively, had a calculated molecular elemental composition C_17_H_20_O_9_ (∆mass = 0.74 ppm) assigned to parent ion [M–H]^–^ with an accurate mass *m*/*z* 367.1037. The MS/MS fragmentation indicated the structure contained a ferulic acid moiety (*m*/*z* 193.1). According to the elemental composition and fragmentation pattern, it was speculated that these two features belonged to the feruloylquinic acid family [41]. However, authentic commercial standards were not available, thus 1D and 2D NMR analysis was required for positive identification (see Supplemental Materials, Figures S2 and S3). Compounds **4** and **5** were isolated from the coffee samples through multiple dimensions of LC fractionation as was described in Section 3.6, with >90% purity for NMR analysis.

The ^1^H NMR data showed similar key chemical shifts and coupling constants, indicating structural isomerism between these compounds. Compound **4** showed three aromatic signals at δ 7.20 (d, *J* = 2.0 Hz, 1H), δ 7.08 (dd, *J* = 8.1, 2.5 Hz, 1H), and δ 6.81 (dd, *J* = 8.2, 2.5 Hz, 1H), indicating the trisubstituted ring moiety. Also, two olefinic signals at δ 7.66 (d, *J* = 15.9 Hz, 1H), and δ 6.40 (d, *J* = 15.9 Hz, 1H), revealed the *E* geometry in the double bond in the vinylcatechol group. The key signals attributed to three carbinolic methines at δ 5.36 (dt, *J* = 6.1, 3.6 Hz, 1H), δ 4.12 (td, *J* = 8.2, 3.9 Hz, 1H), and δ 3.69 (dd, *J* = 7.8, 3.3 Hz, 1H) confirmed the caffeic acid moiety. All ^1^H and ^13^C NMR data analysis of compound **4** and the match with the data reported in the literature allowed for the identification of this compound as 3-O-feruloylquinic acid [42]. Likewise, compound **5** presented similar key NMR signals, scilicet, three aromatic signals located at δ 7.20 (s, 1H), δ 7.08 (dd, *J* = 8.2, 2.0 Hz, 1H), and δ 6.81 (dd, *J* = 8.8, 2.6 Hz, 1H), two olefinic signals resonating at δ 7.63 (d, *J* = 15.9 Hz, 1H) and δ 6.39 (d, *J* = 15.9 Hz, 1H), indicating *E* configuration, and the same three carbinolic methines groups located at δ 5.46 (ddd, *J* = 11.6, 9.9, 5.0 Hz, 1H), δ 4.12 (q, *J* = 3.3 Hz, 1H), and δ 3.66 (dd, *J* = 9.8, 3.1 Hz, 1H). All ^1^H and ^13^C NMR data analysis of compound **5** matched the data reported in the literature for compound 5-O-feruloylquinic acid [42], allowing the structural identification and confirming the structural isomerism between compounds **4** and **5**.

### 2.4. Targeted Analysis of Hydrophilic Coffee Acids

The reported decrease in coffee pH during storage indicated the importance of monitoring acidic compounds. In the current study, it was anticipated that sample clean-up losses and limited reverse-phase chromatographic separation resulted in some hydrophilic acids being not adequately included in the untargeted chemical profiling and the statistical modeling. Therefore, a targeted analysis of well-known relatively abundant hydrophilic acids in the coffee samples was also conducted. Acids are an important chemical component of coffee, which account for about 6% of the roasted coffee beans’ weight and contribute to the pH and titratable acidity in the coffee brew [36]. The more abundant hydrophilic acids in coffee, namely citric acid, quinic acid, malic acid, and phosphoric acid [43,44] were monitored by targeted analysis. Quantitative and statistical analysis of these four compounds reported that only quinic acid presented significant concentration differences (*p* < 0.05) between non-aged and aged samples, which is reported in Table 2. Consequently, quinic acid (compound **6**, Table 2) was also selected, in addition to the positively correlated predictive chlorogenic acid compounds **1**–**5,** for further investigation.

### 2.5. Quantification of Positive Correlated Predictive Compounds in RTD Coffee Samples

The RTD coffee samples made from different coffee species and processing conditions exhibited similar trends in chemical and flavor change during storage; therefore, the Arabica nitrogen-flushed RTD coffee was selected as a representative sample for the following quantification and sensory recombination testing. The concentrations of compounds **1**–**5** were quantified in the non-aged and 4-month aged samples, shown in Table 2.

The concentrations of the acidic compounds **1**–**5** significantly (*p* < 0.05) increased during storage and are in agreement with the noted decrease in pH for RTD coffee samples during storage. Quinic acid showed the largest absolute concentration change by increasing 169 mg/L over 4 months, while 3-caffeoylquinic acid presented the largest percentage change of 47%. An increase in quinic acid concentration after brewing has been reported, which is generally attributed to the breakdown from chlorogenic acids and the hydrolysis of quinic acid lactones [37,44,45]. In a similar way, hydrolysis of the intramolecular ester bond in chlorogenic acid lactones has also been observed in the coffee brew [46,47,48], which to some extent explained the increase in chlorogenic acid concentration including caffeoylquinic acids (CQAs) and feruloylquinic acids (FQAs). More recently, it was reported that quinic acid and chlorogenic acids are incorporated into low molecular weight coffee brew melanoidins during the roasting process [49,50,51,52]. The subsequent release of acids from melanoidins during storage may explain in part the acidification of RTD coffee during storage.

Subsequently, the influence of compounds **1**–**6** on RTD coffee acidification during storage was evaluated. Compounds **1**–**6** were added to the non-aged Arabica nitrogen-flushed sample to match the concentration of the 4-month aged sample (Table 2). The addition of compounds **1**–**6** resulted in the pH decreasing from 4.92 to 4.79, which accounted for 81% of the total pH decrease compared to a 4-month aged sample at pH 4.76. Therefore, the main compounds that impacted pH change during storage were identified.

### 2.6. Sensory Recombination of Identified Acids in RTD Coffee Samples

**Figure 3:**
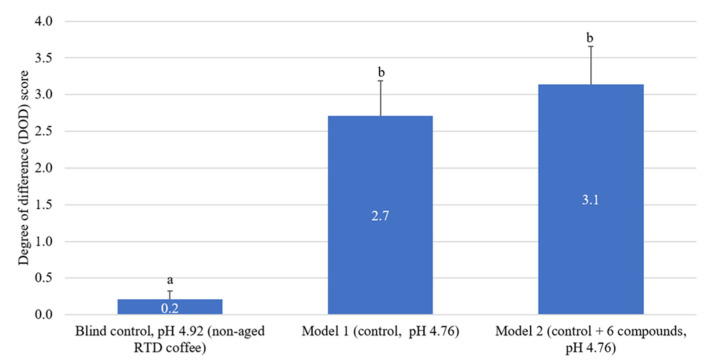
Mean degree of difference (DOD) scores with standard error for blind control and recombination model 1 (control, pH adjusted) and model 2 (control, pH adjusted with the addition of 81.5, 58.5, 44.3, 18.5, 9.3, and 168.9 mg/L of 3-caffeoylquinic acid, 5-caffeoylquinic acid, 4-caffeoylquinic acid, 3-O-feruloylquinic acid, 4-O-feruloylquinic acid and quinic acid, respectively); different letters represent significant differences in DOD scores according to 1-sided Dunnett’s test (*p* < 0.05); *n* = 14, DOD scores ranged from 0–10.

The sensory impact of the highly predictive positively correlated acidic compounds **1**–**5**, as well as the additional targeted acidic compound **6** (Table 2), were further investigated. The DOD scores of two aged RTD coffee recombination models were compared to the non-aged RTD coffee sample. Model 1 was the control sample that was pH adjusted (HCl) to mimic the RTD coffee after 4 months storage, while model 2 was also pH adjusted but additionally contained the higher concentrations of compounds **1**–**6** as reported in the aged coffee sample (Table 2). As shown in Figure 3, the DOD score of the blind control sample was rated as 0.2, indicating the good performance of the trained panelists (negligible detectable difference). Both models 1 and 2 with DOD scores of 2.7 and 3.1, respectively, were significantly different from the blind control sample (non-aged) at *p* < 0.05. The DOD scores for models 1 and 2 were not significantly different from each other and corresponded to a little difference on the DOD scale.

Results from model 1 (Figure 3) indicated that the decrease in pH that occurred during storage significantly changed the overall flavor of the RTD coffee sample. Panelists (10 out of 14) mentioned that sourness was the primary reason for the difference between the control sample and model 1. The initial DOD sensory evaluation for the 4-month aged Arabica nitrogen-flushed RTD coffee samples received a DOD score of 6.6, while recombination model 1 received a DOD score of 2.7, accounting for about 40% of the coffee samples DOD score. In general, the pH of Arabica coffee brew ranges from 4.85 to 5.13 [53,54], and a pH of 4.8 or higher is considered a critical value for acceptable coffee quality [37]. A change in coffee brew pH of 0.1 units has resulted in significant differences in perceived sourness [55]. In the current study, a decrease in coffee pH of 0.16 units (4.94 to 4.76) was reported to significantly impact the sensory profile of the sample. Similar to the sensory DOD analysis of the non-aged to aged coffee samples, panelists indicated that sourness was one of the primary drivers for overall flavor changes in the recombination model 1 (Figure 3). These results indicated that sourness development in the RTD coffee was caused by the decrease in pH over time, which significantly contributed to the overall flavor changes during storage.

In addition to the impact of pH change on flavor stability of RTD coffee during storage, the influence of the increased concentration of the weak acids on sensory DOD scores was also evaluated in model 2 (Figure 3). Sourness in coffee has been related to both the pH value and total acidity [44]. Model 2 was not reported to be significantly different from model 1 (Figure 3) in DOD score, indicating the increased acid concentration during storage did not impact the flavor stability due to a higher total acidity content but rather by the direct change in pH (Table 2).

Although the panelists indicated sour was the primary difference observed between the samples, in addition to sourness, quinic acid, and chlorogenic acids have been associated with coffee flavor attributes such as bitterness, astringency, and lingering aftertaste in past studies [9,56,57]. Quinic acid was reported to exhibit an aspirin-like bitter taste at a threshold level of 10 mg/L [58]. Moreover, quinic acid was found to be associated with astringency perception and lingering aftertaste in cranberry juice [59] and fruit pulps [60]. In the current study, comparing the increase of quinic acid from 1259 mg/L to 1429 mg/L during storage (Table 2) suggests this compound may have contributed to the overall flavor of RTD coffee (bitterness, astringency). However, the increased concentration of quinic acid during storage was not found to increase the DOD beyond the impact of pH change (Figure 3). Compounds **1**–**5** (CQAs and FQAs) belong to the chlorogenic acid family, which is associated the sourness, astringency, and other sensory attributes of coffee brew [61,62,63]. Similarly, the increased amount of these chlorogenic acids in the RTD coffee during storage was not shown to impact the DOD beyond the influence of pH (Figure 3). The higher chlorogenic acid content in coffee has been shown to be associated with less bitterness [28,64] and more astringency [65,66]. However, the higher concentrations of quinic acid and chlorogenic acids observed in the aged samples (Figure 3) did not significantly impact the reported DOD beyond the impact of sample pH.

Changes in the aroma were also reported in the aged samples; however, they were less frequently noted than taste (sour). This study focused specifically on the non-volatile flavor changes. However, changes in the aroma volatility and stability would be expected. Some potent well-known coffee odorants such as 2-furfurylthiol and 3-mercapto-3-methylbutyl esters have shown pH dependence in RTD coffee drinks [17,67].

Comparing the sensory recombination DOD score for model 2 of 3.1 (Figure 3) with the 4-month aged coffee sample (6.6), it can be seen that increasing concentrations of chlorogenic acids and quinic acid over time contributed to the overall flavor changes in the Arabica nitrogen-flushed RTD coffee samples during storage. As discussed, the increased concentrations of chlorogenic acids could be partially explained by the hydrolysis of their corresponding chlorogenic acid lactones.

## 3. Materials and Methods

### 3.1. Chemicals and Materials

Optima-grade formic acid, acetonitrile, methanol, acetone, and food-grade hydrochloric acid (HCl) were purchased from Fisher Scientific (Waltham, MA, USA). Quinic acid, methylparaben, deuterated methanol, and deuterated water were purchased from Millipore Sigma (Burlington, MA, USA). 3-Caffeoylquinic acid (3-CQA), 4-caffeoylquinic acid (4-CQA), and 5-caffeoylquinic acid (5-CQA) were purchased from BOC Sciences (Shirley, NY, USA). Nanopure water was purified through Barnstead Nanopure Diamond Water Purification System (Thermo Fisher, Dubuque, IA, USA). Leucine enkephalin was purchased from Waters Co. (Milford, MA, USA). Organic green Arabica and Robusta coffee beans were purchased from local suppliers (Columbus, OH, USA).

### 3.2. Ready-to-Drink (RTD) Coffee Samples

The Arabica and Robusta green coffee beans were first light-roasted in line with a roast color of 100 CTN (Jupiter Tangential Roaster, Probat, Emmerich, Germany) and then ground into coarse particles with a size of d’ = 2.4 mm. Deoxygenated and deionized water at 85 °C was used to make coffee brews in a 1:10 coffee-to-water ratio, which was prepared using a French press coffee maker with a 0.037 mm mesh screen. After steeping for 5 min, coffee brews were decanted and sealed in steel cans (approximately 180 mL). To examine the effect of oxygen presence on RTD coffee flavor during storage, two processing conditions were applied to sample preparation. Specifically, air-headspace RTD coffee samples were prepared in an open-air condition while nitrogen-flushed RTD coffee samples were prepared in a nitrogen glove box. All sealed cans were retorted at 125 °C for 5 min for sterilization. Following sterilization, the samples were evenly divided into four different groups: group 1 was fresh sample (hereafter non-aged samples) stored in a −40 °C freezer while groups 2, 3, and 4 were stored in a 30 °C Barnstead Lab-line incubator (Lab-line/Barnstead, Dubuque, IA, USA) for 1, 2, and 4 months, respectively. After storage, all samples were transferred to a −40 °C freezer. The total solid content of RTD coffee samples was normalized to 1.6%. In total, there were 16 independent samples (*n* = 16, 2 coffee species × 2 processing conditions × 4 storage time points) for instrumental and sensory analysis (see Supplemental Materials, Figure S4).

### 3.3. Sensory Evaluation by the Degree of Difference (DOD) Test

Sensory evaluation of the overall flavor differences between non-aged and aged RTD coffee at 1, 2, and 4 months was carried out using a Degree of Difference (DOD) test [68]. Fourteen trained panelists (6 males and 8 females, aged 23 to 44 years) from the Ohio State University participated in the study for a total of 4 sessions over 4 days. In each session, only one RTD coffee variety (Arabica air-headspace, Arabica nitrogen-flushed, Robusta air-headspace, Robusta nitrogen-flushed) was evaluated. Before panelists arrived, non-aged and each aged RTD coffee samples were pre-heated to 60–65 °C and kept in different insulated air pots to maintain serving temperature. When panelists arrived, a set of samples including a control sample (non-aged) and 4 test samples (1 non-aged as blind control and 3 aged samples from 1, 2, and 4 months) was presented to panelists in 3 oz black ceramic cups (approximately 70 mL for each sample). The order of presenting test samples was randomized across panelists. To compare each test sample against the control, panelists were asked to taste the control first and then the test sample; and to place the cup on a large, printed DOD scale, which helped visualize the size of the flavor difference between the control and the test sample. The DOD scale ranged from 0 to 10 points, with a description below the numbers explaining the size of difference: 0 corresponding to ‘none’, 1 and 2 corresponding to ‘very little’, 3 and 4 corresponding to ‘little’, 5 and 6 corresponding to ‘medium’, 7 and 8 corresponding to ‘large’, and 9 and 10 corresponding to ‘extreme’. Overall, there were four comparisons following the same procedure. Results were entered into Compusense Cloud sensory analysis software (Compusense, Guelph, ON, Canada). Additionally, panelists were encouraged to specifically describe the flavor differences if they considered the test sample different from the control. To eliminate potential flavor carryover, panelists were provided with water and unsalted crackers in between each comparison. Study protocols were approved by the OSU Institutional Review Board (2017H0072).

### 3.4. Ultra-Performance Liquid Chromatography-Mass Spectrometry (UPLC-MS) Chemical Profiling

Solid-phase extraction (SPE) of non-volatile analytes from RTD coffee samples was carried out using an Oasis PRiME HLB 96-well plate cartridge (Waters Co., Milford, MA, USA). First, 16 independent samples (600 µL) were diluted with nanopure water (400 µL) and loaded onto cartridge; then 5% methanol/water (*v*/*v*) (500 µL) was used to remove salts and highly polar compounds; last, 95% acetonitrile/water (*v*/*v*) (200 µL) was used to elute analytes retained on the cartridge. Prior to UPLC-MS analysis, the eluent was further diluted with nanopure water in a 1:4 ratio. Following the same process, a quality control (QC) sample was prepared by mixing an equal amount (1 mL) of all RTD coffee samples. A Hamilton MicroLab Star Plus Liquid Handling System (Hamilton Robotics, Reno, NV, USA) was used to automatically perform the SPE process.

Ultra-performance liquid chromatography coupled with a time-of-flight (Q-ToF) mass spectrometer (Waters Acquity H-Class quaternary solvent manager with Waters Synapt G2-S mass spectrometer, Waters Co., Milford, MA, USA) was employed to collect non-volatile chemical fingerprints of RTD coffee samples. Two microliters of RTD coffee analytes was injected into a reverse-phase Cortecs C18+ column (1.6 µm, 2.1 × 100 mm, Waters Co., Milford, MA, USA) held at 40 °C. The mobile phase was composed of (A) water, (B) acetonitrile, and (C) 5% formic acid in water (*v*/*v*) at a flow rate of 0.5 mL/min. The gradient started with holding 5% B for half a minute (0–0.5 min), increased to 50% B in 10.5 min (0.5–11 min), further increased to 95% B in 1.5 min (11–12.5 min), then held at 95% B for 1.5 min (12.5–14 min), and went back to initial conditions. Solvent C was constantly held at 2%. The mass spectrometer was operated in negative electrospray ionization mode with following parameters: capillary voltage = 2.5 kV, cone voltage = 35 V, source temperature = 120 °C, desolvation temperature = 450 °C, cone gas flow = 120 L/h, desolvation gas flow = 800 L/h, and nebulizer pressure = 6.0 bar. Q-ToF analyzer was set to scan a mass range of 50 to 1200 *m*/*z* with 0.3 sec scan time. Leucine enkephalin (*m*/*z* 556.2771) was used as an internal standard for mass correction throughout the analysis.

All 16 independent RTD coffee samples with 2 biological and 2 technical replicates were injected into the UPLC-MS in randomized order. To monitor analytical performance, a water blank, a column standard (mixture of 4 parabens), and the QC sample were injected after running every 10 samples.

### 3.5. Multivariate Statistical Analysis (MVA)

Chromatographic and spectral data were converted into statistical variables by deconvolution, ion extraction, and integration using Progenesis QI software (Nonlinear Dynamics, Durham NC). Each chemical feature was reported as a retention time-mass/charge ratio (RT_m/z) with ion abundance. Chemical features exported from Progenesis QI were further processed in the R program (version 3.5.2, R Foundation, Vienna, Austria) to filter out noise based on ion intensity threshold (>500 counts) and coefficient of variance between replicates (<30%). Multivariate data analysis was performed with two RTD coffee biological replicates by using SIMCA-P+ 14.1 (Umetrics, Umeå, Sweden). Principal component analysis (PCA) and orthogonal partial least squares (OPLS) models were generated using Pareto scaling. In the OPLS model, DOD scores of RTD coffee samples were assigned as Y variables, while chemical features (RT_*m*/*z* by ion abundance) were assigned as X variables. The predictive variable of importance (VIPpred) scores and S–plot were subsequently generated to select highly significant predictive chemical features. The features discussed in the current paper were referred to as positively correlated features because their concentrations in RTD coffee samples increased as the degree of flavor differences increased over time.

### 3.6. Off-Line Multidimensional Preparative-Liquid Chromatography/Mass Spectrometry (Prep-LC/MS) Fractionation

According to the model’s predictive ability, top chemical features were selected, isolated, or purchased to investigate their impact on the flavor stability of RTD coffee during storage in a sensory study. Commercial standards for 3-caffeoylquinic acid, 4-caffeoylquinic acid, and 5-caffeoylquinic acid were purchased from BOC Sciences (Shirley, NY, USA) whereas 3-O-feruloylquinic acid and 5-O-feruloylquinic acid, chemical features (RT_*m*/*z*) 3.59_367.1 and 4.29_367.1, respectively, were isolated from the coffee brew as detailed below (see Supplemental Materials, Figure S5).

Three hundred grams of fresh coffee grounds was added to 3 L of 80% methanol/water (*v*/*v*). The mixture was stirred at room temperature for 12 h and then filtered through a Whatman grade 4 filter paper (GE Healthcare, Buckinghamshire, UK) and a 5 kDa ultrafiltration membrane (Millipore Sigma, Burlington, MA, USA). Solid-phase extraction (SPE) was performed to clean up filtered coffee. Specifically, 200 mL filtered coffee was loaded onto an Oasis HLB 6-g bed cartridge (Waters, Milford, MA, USA). Then, 100 mL of 5% methanol/water (*v*/*v*) was used to wash the cartridge, and 100 mL of 95% methanol/water (*v*/*v*) was used to elute analytes from the cartridge. The SPE process was repeated for several cartridges to increase extraction yield. The eluent was subsequently freed from the solvent using a Rocket Synergy Purge (Genevac, Ipswich, UK) and lyophilized. The lyophilized sample was reconstituted to approximately 500 mg/L in 20% methanol/water (*v*/*v*), filtered through a PTFE 0.45 μm filter, and then injected into the Prep LC-MS fractionation system (Waters 2545 binary pump and TQD mass spectrometer coupled with 2767 fraction collector). First dimension isolation was achieved using an Xbridge Prep C18 column (5 µm, 50 mm × 50 mm, Waters Co., Milford, MA, USA). The mobile phase was maintained at a 100 mL/min flow rate using a binary solvent system of 0.1 % formic acid in water (A) and methanol (B). The gradient was set as follow: 0–0.5 min, 5% B; 0.5–1.5 min, 5–27% B; 1.5–5.5 min, 27% B; 5.5–8.5 min, 27–50% B; 8.5–10 min, 50–95% B; 10–13 min, 95%; 13.01–15 min, 5% B. First-dimension fractions were pooled, freed from solvent, and lyophilized before reconstituting to approximately 500 mg/L in 10% methanol and filtering through a 0.45-μm filter.

To achieve better purity, second dimension HPLC fractionation was performed on an Atlantis T3 OBD column (5 µm, 50 mm × 250 mm, Waters Co., Milford, MA, USA) using a mobile phase consisting of (A) water with 0.1% formic acid and (B) acetone with 0.1% formic acid. The gradient was set at 100 mL/min as follows: 0–0.5 min, 5% B; 0.5–1 min, 5–20% B; 1–30 min, 20% B; 30–31 min, 20–95% B; 31–34 min, 95% B; 34.01–37 min, 5% B. The same column and gradient were applied to both features. The second-dimension fractions were handled using the same protocol as the first-dimension fractions.

To achieve purity greater than 90%, a third-dimension fractionation was performed utilizing an Xselect CSH Phenyl-Hexyl OBD prep column (5 µm, 10 × 250 mm, Waters Co., Milford, MA, USA) on a semi-prep scale. The mobile phase consists of (A) water with 0.1% formic acid and (B) acetone with 0.1% formic acid at a flow rate of 7 mL/min. The gradient was optimized as follows: 0–1 min, 12% B; 1–23 min, 12–20% B; 23–25 min, 20–95% B; 25–27.5 min, 95%; 27.51–30 min, 5% B. The same column and gradient were applied to both features.

The TQD mass spectrometer was operated under negative ESI mode using the following settings: capillary voltage = 2.5 kV, cone voltage = 30 V, source temperature = 150 °C, and desolvation gas temperature = 400 °C. The time-based collection was applied to collect the first-dimension fractions within the retention time range of each targeted feature. Mass-triggered collection under single ion monitoring (SIR) mode was used to collect the 2nd and 3rd dimension fractions. After each collection, pooled fractions were injected to Synapt G2-S UPLC-QToF-MS (Waters Co., Milford, MA, USA) to ensure accurate collection of targeted chemical features. The isolate purity was initially calculated based on total ion chromatogram peak area under both positive and negative ESI modes (>90%). The chemical isolates were further analyzed by NMR analysis, which verified > 94% purity. The high purity isolates were subsequently utilized for quantification and sensory recombination testing.

### 3.7. Quantification by Ultra-Performance Liquid Chromatography-Tandem Mass Spectrometry (UPLC–MS/MS)

The concentrations of compounds **1**–**6** (Table 2) were quantified in the non-aged and 4-month aged Arabica nitrogen-flushed RTD coffee samples using a UPLC Waters Acquity H-Class system coupled to a TQS mass spectrometer (Waters Co., Milford, MA, USA). Quantification was carried out with 5-point standard addition calibration curves (in triplicate) and displayed good linearity of all the compounds (R^2^ > 0.98). Sample preparation was performed by diluting 600 µL RTD coffee samples with 400 µL nanopure water with the addition of 100 mg/L methylparaben as an internal standard. Quantitative analysis was conducted using an Acquity H-class UPLC system (Waters Co., MA., Milford, MA, USA) coupled with a Xevo TQ-S mass spectrometer (Waters Co., MA., Milford, MA, USA) in multiple reaction monitoring (MRM) acquisition mode. Chromatographic separation of targeted compounds was achieved using a Cortecs UPLC T3 column (1.6 µm, 2.1 × 100 mm, Waters Co., Milford, MA, USA). The mobile phase consists of (A) water with 0.1% formic acid (*v*/*v*) and (B) acetone with 0.1% formic acid at a flow rate of 0.5 mL/min. The gradient was optimized as follows: 0–0.5 min, 5% B; 0.5–10 min, 5–15% B; 10–10.5 min, 15–20% B; 10.51–13 min, 95%; 13.01–16 min, 5% B. The concentration of quinic acid was also quantified using the same protocol with modification on the gradient as follows: 0–3 min, 0% B; 3–5 min, 0–40% B; 5.01–7 min, 95% B; 7.01–9, 0% B. The mass spectrometer was operated under negative ESI mode with a capillary voltage of 3 kV, cone voltage of 30 V, desolvation temperature of 550 °C, source temperature of 150 °C, desolvation gas flow of 1000 L/h, and cone gas flow rate of 150 L/h. MRM transition was optimized for each compound and presented in Table 2. MRM transition of methylparaben (internal standard) was monitored as *m*/*z* 150.95 → 91.85 with a cone voltage of 20 V and collision energy of 18 V.

### 3.8. Sensory Validation in RTD Coffee

The non-aged Arabica nitrogen-flushed RTD coffee sample (pH = 4.92) was used as a control sample for the sensory recombination test. A decrease in pH in the RTD coffee sample was observed during storage; hence, two RTD coffee models were prepared to evaluate the effects of the highly predictive chemical features and the pH, separately. The RTD coffee model 1 was prepared by only adding food-grade HCl to reach the pH of an aged sample (pH = 4.76), representing a pH adjusted control sample. The RTD coffee model 2 was prepared by spiking a mixture of compounds **1**–**6** into the control sample to match the concentration of these compounds in a 4-month aged Arabica nitrogen-flushed RTD coffee sample (see Table 2). The addition of compounds **1**–**6** reduced the pH of the non-aged RTD coffee to 4.79, which was further adjusted using food-grade HCl to mimic the pH of an aged sample (pH = 4.76).

Fourteen trained panelists (6 males and 8 females, ages 23 to 44) from the Ohio State University participated in the sensory recombination test. The RTD coffee control sample, model 1, and model 2 were kept in a hot water bath to maintain serving temperature (60 °C to 65 °C). Panelists were served 5 mL samples in 1 oz black cups. Panelists were asked to evaluate 3 pairs of RTD coffee samples following the same DOD protocol described in Section 3.3. In order to maintain serving temperature, panelists were given one pair of samples at a time; each pair consisted of a control sample and a test sample (control sample as blind control, model 1, or model 2). The serving order of test samples was randomized and balanced. All data were recorded using Compusense Cloud software (Compusense, Guelph, ON, Canada). Unsalted crackers and water were provided for panelists to cleanse their palate between samples.

### 3.9. Nuclear Magnetic Resonance (NMR)

The two selected chemical features that could not be compared to authentic commercial standards were identified using nuclear magnetic resonance (NMR). NMR analysis was performed on a Bruker Advance III HD Ascend spectrometer equipped with a 5 mm triple resonance observe TXO cryoprobe with z-gradients, operating at 700 MHz for the ^1^H nucleus and 176 MHz for the ^13^C nucleus (Bruker BioSpin, Rheinstetten, Germany). Instruments were calibrated using the residual undeuterated solvent as an internal reference CD_3_OD ^1^H NMR = 3.31 ppm, ^13^C NMR = 49.0 ppm. Deuterated methanol-d4 was used as a solvent to dissolve purified compounds **4** (3.59_367.1) and **5** (4.29_367.1), and NMR data are presented here.

3-*O*-feruloylquinic acid (3.59_367.1): ^1^H NMR (700 MHz, MeOD) 7.66 (d, *J* = 15.9 Hz, 1H), 7.20 (d, *J* = 2.0 Hz, 1H), 7.08 (dd, *J* = 8.1, 2.5 Hz, 1H), 6.81 (dd, *J* = 8.2, 2.5 Hz, 1H), 6.40 (d, *J* = 15.9 Hz, 1H), 5.36 (dt, *J* = 6.1, 3.6 Hz, 1H), 4.12 (td, *J* = 8.2, 3.9 Hz, 1H), 3.90 (s, 3H), 3.69 (dd, *J* = 7.8, 3.3 Hz, 1H), 2.31 (ddd, *J* = 11.6, 6.1, 2.8 Hz, 1H), 2.21 (ddd, *J* = 14.1, 6.9, 3.6 Hz, 1H), 2.10 (d, *J* = 11.6 Hz, 1H), 2.05 (m, 1H). ^13^C NMR (176 MHz, MeOD) δ 175.7, 167.9, 149.4, 147.8, 146.7, 127.9, 124.1, 115.7, 115.0, 111.7, 75.1, 72.6, 70.2, 68.9, 56.4, 37.8, 36.9.

*5-O*-feruloylquinic acid (4.29_367.1): ^1^H NMR (700 MHz, MeOD) δ 7.63 (d, *J* = 15.9 Hz, 1H), 7.20 (s, 1H), 7.08 (dd, *J* = 8.2, 2.0 Hz, 1H), 6.81 (dd, *J* = 8.8, 2.6 Hz, 1H), 6.39 (d, *J* = 15.9 Hz, 1H), 5.46 (ddd, *J* = 11.6, 9.9, 5.0 Hz, 1H), 4.12 (q, *J* = 3.3 Hz, 1H), 3.90 (s, 3H), 3.66 (dd, *J* = 9.8, 3.1 Hz, 1H), 2.28 (ddd, *J* = 12.6, 5.0, 3.0 Hz, 1H), 2.20–2.15 (m, 1H), 2.11 (dt, *J* = 14.3, 3.2 Hz, 1H), 1.97 (dd, *J* = 14.3, 2.9 Hz, 1H). ^13^C NMR (176 MHz, MeOD) δ 182.7, 168.9, 150.5, 149.4, 146.6, 127.9, 124.0, 116.4, 115.9, 111.7, 80.7, 75.2, 73.2, 72.5, 56.4, 39.7, 37.9.

### 3.10. Data Analysis

Statistical analysis was conducted with SPSS Statistics version 25 (IBM, Armonk, NY, USA). DOD scores of each RTD coffee variety were analyzed by one-way ANOVA; when a significant difference was observed (*p* < 0.05), post hoc LSD was performed between all samples and 1-sided Dunnett’s test was used to compare between the blind control (non-aged) and aged samples (1, 2, 4 months aged). Student’s *t*-test was applied to analyze quantification data of non-aged and 4-month aged Arabica nitrogen-flushed RTD coffee samples.

## 4. Conclusions

The comprehensive untargeted LC/MS chemical profiling analysis of RTD coffee during storage revealed among all compounds generated, only acidic compounds impacted the flavor stability. The increase in six specific acidic compounds 3-caffeoylquinic acid, 4-caffeoylquinic acid, 5-caffeoylquinic acid, 3-O-feruloylquinic acid, 5-O-feruloylquinic, and quinic acid, was reported to significantly alter the flavor of RTD coffee. Therefore, the hydrolysis of chlorogenic acid lactones and chlorogenic acids or the release of chlorogenic acids from melanoidins during storage were proposed as key mechanisms of flavor instability. The impact of the six acidulants on coffee flavor was directly related to the change in pH rather than due to the increase in total acidity (amount) of weak acidulants.

## Figures and Tables

**Table 1:** Degree of difference (DOD) scores of ready-to-drink (RTD) coffee samples during storage ^1^.

Sample ^2^	Blind Control, Non-Aged	1 Month	2 Month	4 Month
	(mean ± standard error)			
Arb AHS	0.8 ± 0.3 ^a^	4.6 ± 0.6 ^b^	5.6 ± 0.3 ^bc^	6.6 ± 0.6 ^c^
Arb N2F	1.2 ± 0.3 ^d^	4.4 ± 0.7 ^e^	5.6 ± 0.5 ^ef^	6.6 ± 0.5 ^f^
Rob AHS	0.9 ± 0.2 ^g^	3.9 ± 0.5 ^h^	5.9 ± 0.6 ^i^	5.9 ± 0.5 ^i^
Rob N2F	1.4 ± 0.2 ^j^	5.3 ± 0.5 ^k^	5.3 ± 0.5 ^k^	6.9 ± 0.6 ^l^

^1^ Difference letters represent significant differences in DOD scores according to post hoc LSD test (*p* < 0.05); *n* = 14. ^2^ Arb (Arabica); Rob (Robusta); AHS (air-headspace); N2F (nitrogen-flushed).

**Table 2:** Untargeted and targeted LC/MS compounds in nitrogen flushed Arabica RTD coffee during storage.

Compound No.	Chemical Feature (RT_*m*/*z*)	LC/MS Profiling Method	OPLS VIPpred Score	MRM Transition (Collision Energy)	Compound Identity	Compound Concentration (mg/L) ^i^		Sample Concentration Difference (mg/L)	% Change (Concentration)
						Non-aged RTD coffee	4-month aged RTD coffee		
1	2.68_353.1	Untargeted	3.7	353.1 → 191.1 (20)	3-caffeoylquinic acid	172.9 ^a^	254.4 ^b^	81.5	47.1
2	3.22_353.1	Untargeted	5.6	353.1 → 179.0 (18)	5-caffeoylquinic acid	207.8 ^a^	266.6 ^b^	58.8	28.3
3	3.36_353.1	Untargeted	4.7	353.1 → 191.1 (18)	4-caffeoylquinic acid	165.2 ^a^	209.5 ^b^	44.3	26.8
4	3.59_367.1	Untargeted	4.1	367.1 → 134.0 (34)	3-O-feruloylquinic acid	87.8 ^a^	106.3 ^b^	18.5	21.1
5	4.29_367.1	Untargeted	4.5	367.1 → 191.0 (16)	5-O-feruloylquinic acid	56.9 ^a^	66.2 ^b^	9.3	16.3
6	n/a ^ii^	Targeted	n/a ^ii^	191.1 → 84.9 (22)	Quinic acid	1258.9 ^a^	1427.8 ^b^	168.9	13.4

^i^—Different letters indicate significant differences in compound concentration according to Student’s *t*-test (*p* < 0.05). ^ii^—not available.

## Data Availability

Not applicable.

## Supplementary Materials

The following supporting information can be downloaded at: https://www.mdpi.com/article/10.3390/molecules27072120/s1, Figure S1: Scheme of chemical profiling, Figure S2: Scheme of fractionation of highly predictive features, Figure S3: ^1^H NMR experiments of 3-*O*-feruloylquinic acid, Figure S4: ^1^H and ^13^C NMR experiments of 5-*O*-feruloylquinic acid, Figure S5: MS/MS fragmentation of (A) feature 2.68_353.1, 3-caffeoylquinic acid, (B) feature 3.22_353.1, 5-caffeoylquinic acid, (C) feature 3.36_353.1, 4-caffeoylquinic acid.

## Sample Availability

Samples are not available from the authors.
