# Supplementary material for: Identification of Non-Volatile Compounds Generated during Storage That Impact Flavor Stability of Ready-to-Drink Coffee
Source: Molecules. 2022 Mar 25;27(7):2120. doi: 10.3390/molecules27072120 (PMC9000708; doi:10.3390/molecules27072120)
Supplement: Supplementary file 1 [file molecules-27-02120-s001.zip › molecules-1582440-supplementary.pdf]

Identification of Non-Volatile Compounds Generated during Storage That Impact Flavor Stability of Ready-to-Drink Coffee

Supplemental Material

Hao Lin, Edisson Tello, Christopher T. Simons and Devin G. Peterson

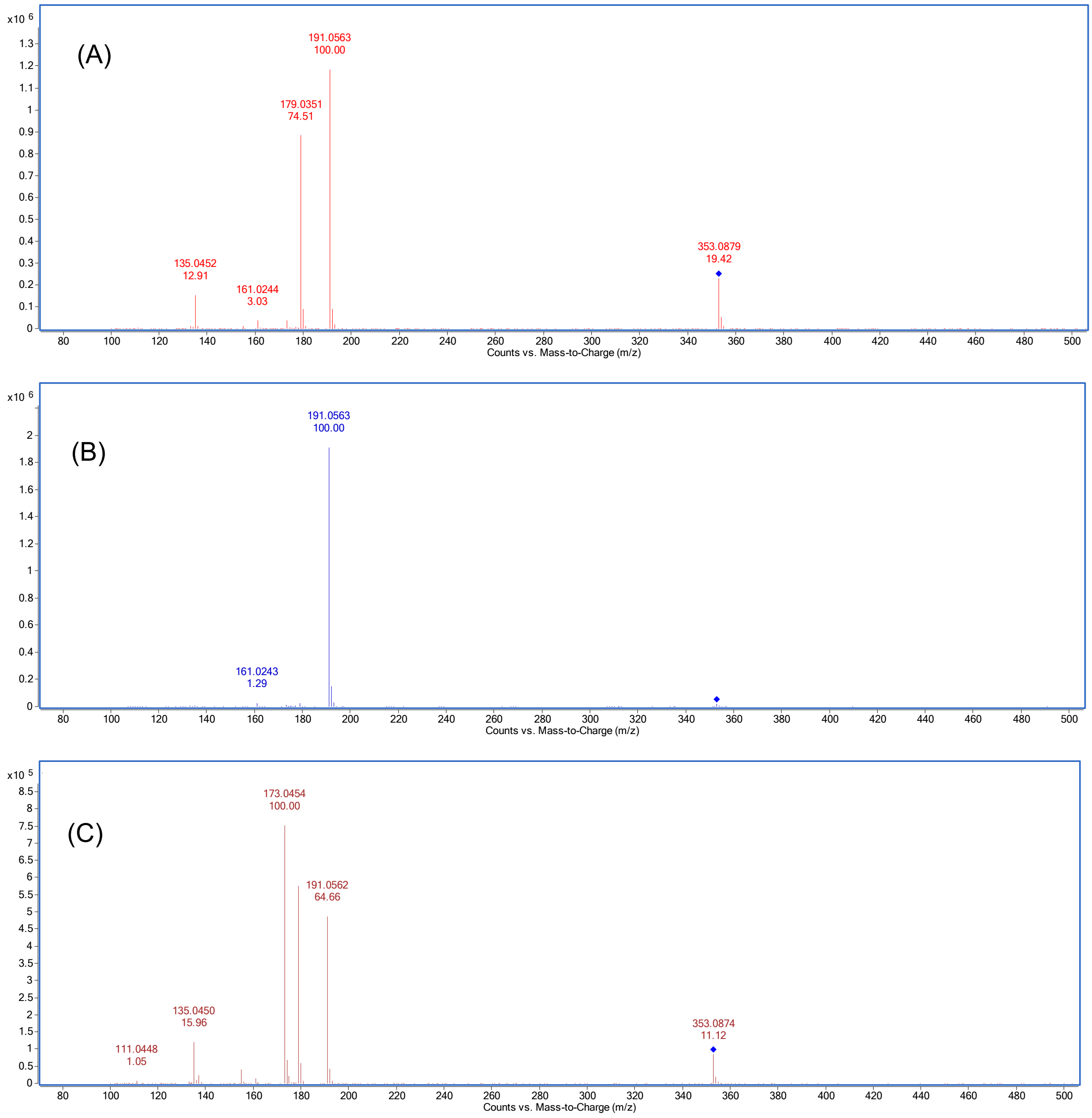

Figure S1. MS/MS fragmentation of (A) feature 2.68_353.1, 3-caffeoylquinic acid, (B) feature 3.22_353.1, 5-caffeoylquinic acid, (C) feature 3.36_353.1, 4-caffeoylquinic acid

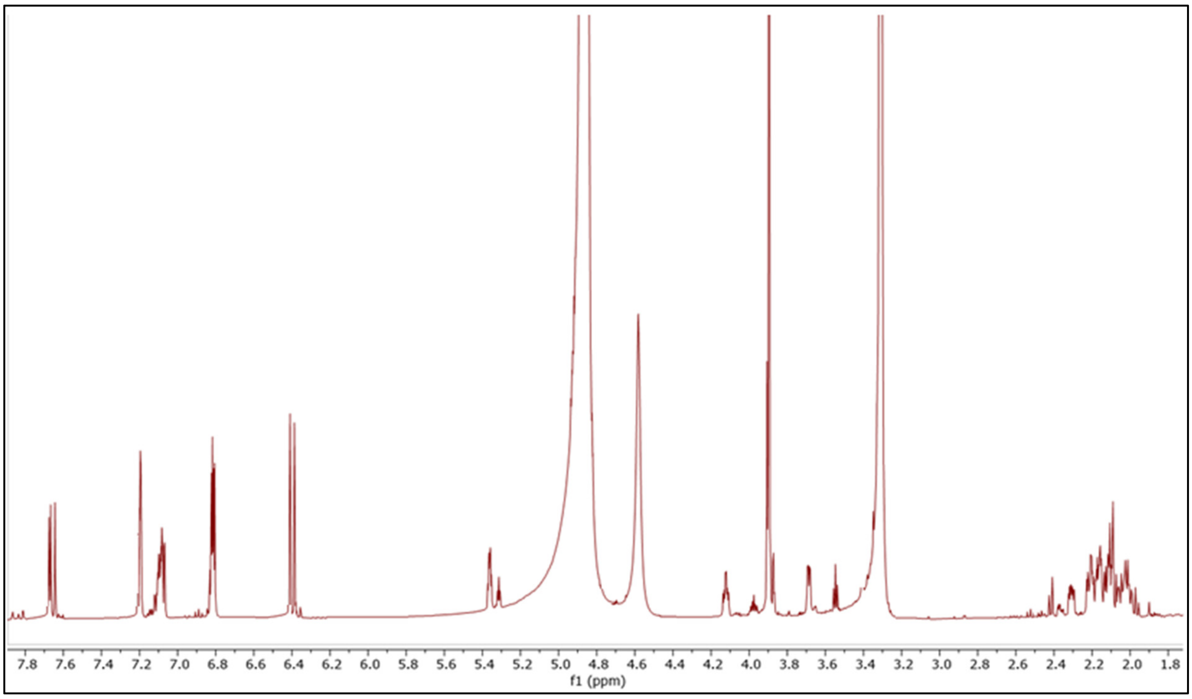

Figure S2. ^{1}H NMR experiments of 3-*O*-feruloylquinic acid.

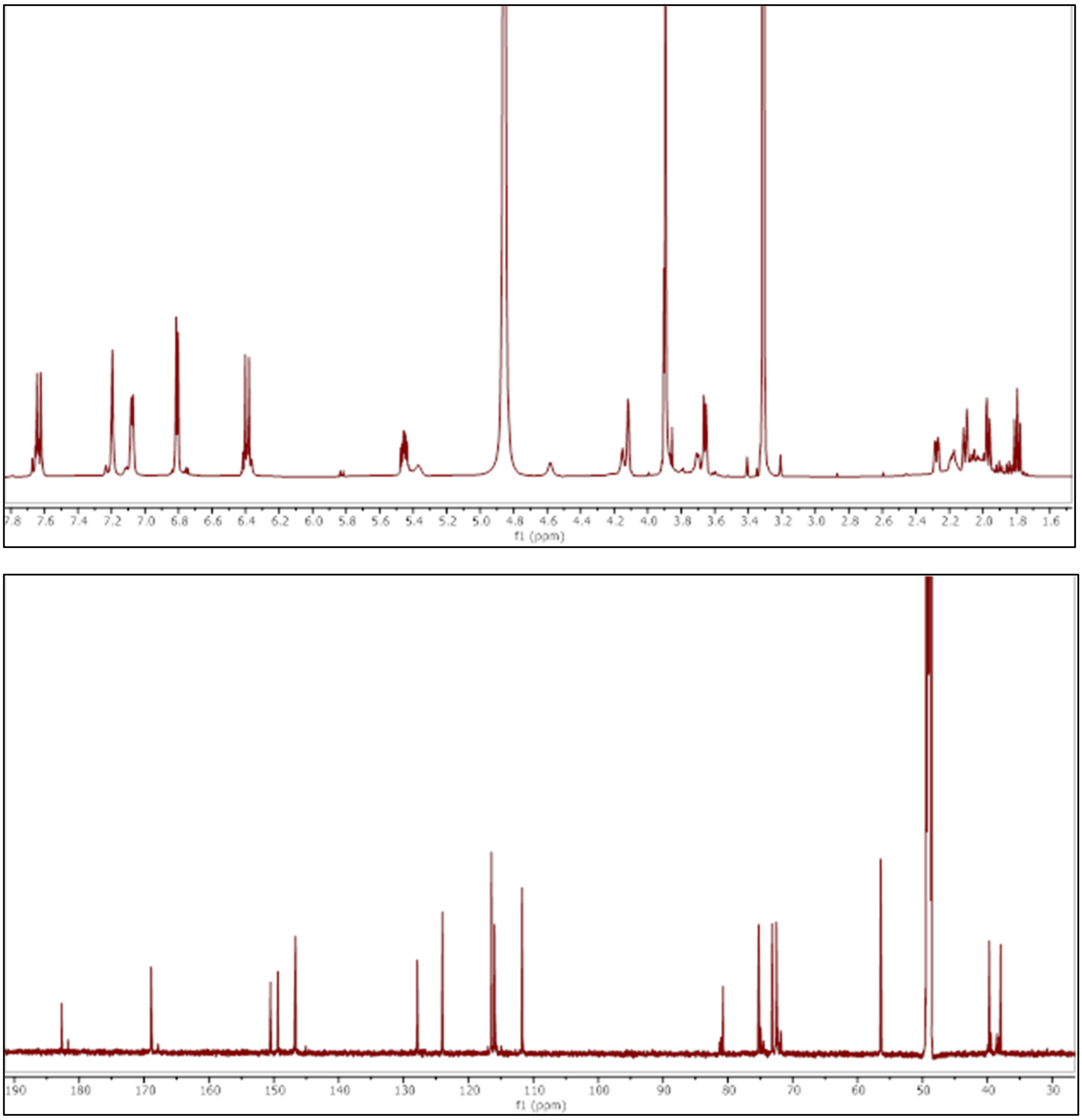

Figure S3. ^{1}H and ^{13}C NMR experiments of 5-*O*-feruloylquinic acid.

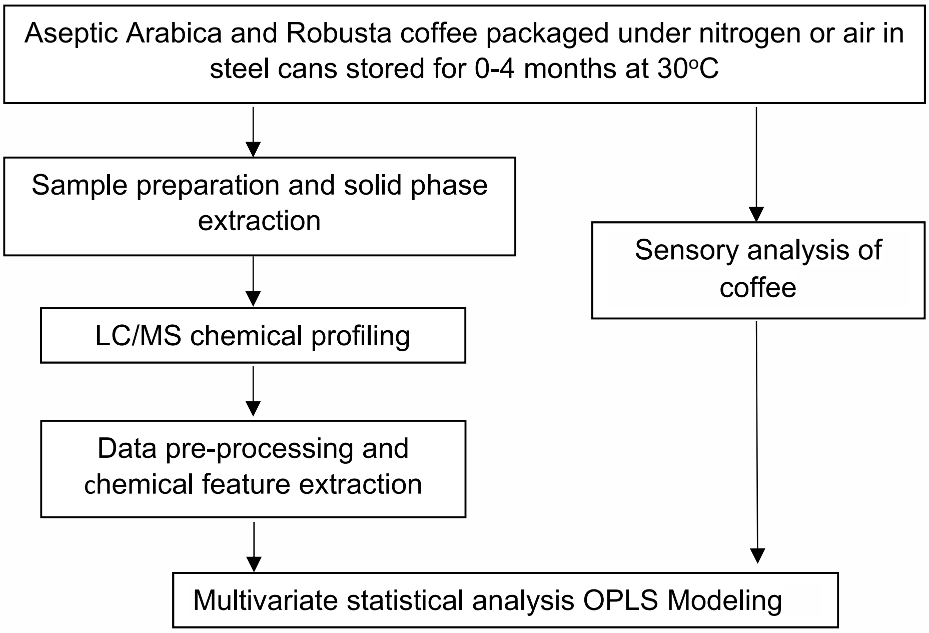

Figure S4. Scheme of chemical profiling and data analysis

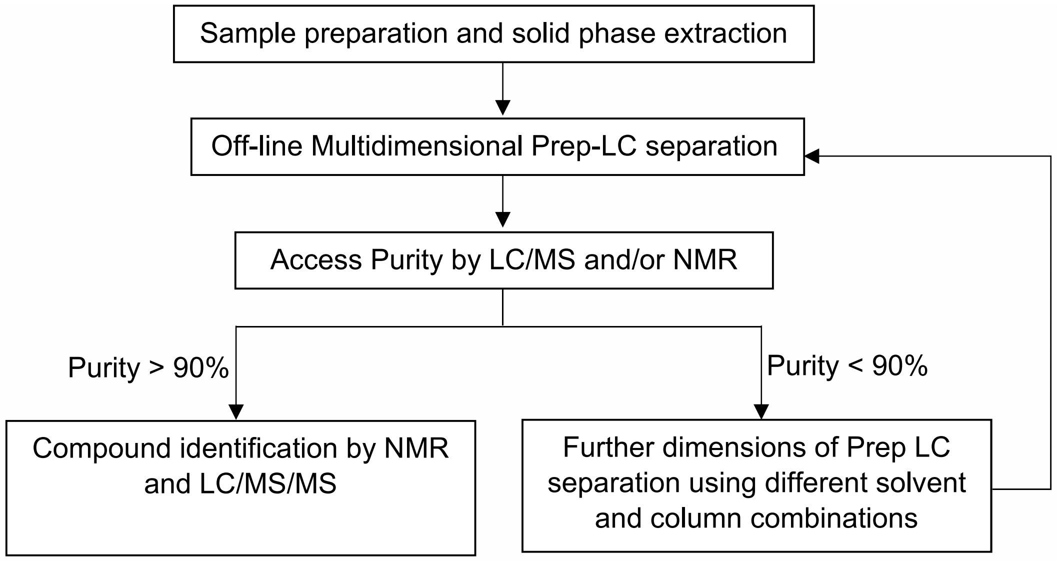

Figure S5. Scheme of fractionation of highly predictive features
